# A Reconfigurable Readout Integrated Circuit for Heterogeneous Display-Based Multi-Sensor Systems

**DOI:** 10.3390/s17040759

**Published:** 2017-04-03

**Authors:** Kyeonghwan Park, Seung Mok Kim, Won-Jin Eom, Jae Joon Kim

**Affiliations:** School of Electrical and Computer Engineering, Ulsan National Institute of Science and Technology, 44919 Ulsan, Korea; khpark@unist.ac.kr (K.P.); nailmong@unist.ac.kr (S.M.K.); avella@unist.ac.kr (W.-J.E.)

**Keywords:** heterogeneous multi-sensor functions, alternate sampling ADC, reconfigurable readout, touch sensor, environment sensors, electrocardiogram, body impedance

## Abstract

This paper presents a reconfigurable multi-sensor interface and its readout integrated circuit (ROIC) for display-based multi-sensor systems, which builds up multi-sensor functions by utilizing touch screen panels. In addition to inherent touch detection, physiological and environmental sensor interfaces are incorporated. The reconfigurable feature is effectively implemented by proposing two basis readout topologies of amplifier-based and oscillator-based circuits. For noise-immune design against various noises from inherent human-touch operations, an alternate-sampling error-correction scheme is proposed and integrated inside the ROIC, achieving a 12-bit resolution of successive approximation register (SAR) of analog-to-digital conversion without additional calibrations. A ROIC prototype that includes the whole proposed functions and data converters was fabricated in a 0.18 μm complementary metal oxide semiconductor (CMOS) process, and its feasibility was experimentally verified to support multiple heterogeneous sensing functions of touch, electrocardiogram, body impedance, and environmental sensors.

## 1. Introduction

Display technology innovations have been focused primarily on their resolution and size, but another remarkable trend is that they are increasingly embedding more different functions such as touch sensors [1]. Among various touch sensors, the mutual-capacitance touch-sensing method has been popular, especially in smartphone applications, and its derivative studies have enhanced touch sensitivity and immunity against noise and interference [2,3]. Recent touch screen panels (TSPs) have become integrated inside display panels. Moreover, there have been recent efforts to implement the fingerprint recognition function on the TSP [4,5]. In flexible or wearable application fields, there have been device-based trials to embed different kinds of signal acquisitions, including bio-signal, respiration and human movements, through various types of sensors [6,7]. Considering these different research trends, this work tries to propose a kind of display-based multi-sensor interface that can embed various sensors from heterogeneous fields. That is, in addition to the inherent touch function of the TSP, two different heterogeneous interfaces of physiological and environmental sensors are proposed. For compact implementation that supports various heterogeneous sensors effectively, its common readout integrated circuit (ROIC) is required to have a reconfigurable interface structure. Additionally, it needs to provide proper remedies against noises and motion that its inherent TSP-based operation accompanies. 

Accordingly, a TSP-based multi-sensor interface is proposed to support various heterogeneous sensors including electrocardiogram (ECG), body impedance (BI) [8,9], and also environmental sensors that are mostly resistive or capacitive [10,11]. In order to accommodate these heterogeneous sensors, the ROIC is designed to have a reconfigurable structure that consists of an amplifier-based signal path and an oscillator-based signal path. The amplifier-based path provides two sensor interfaces for touch and ECG, and the oscillator-based path gives reconfigurable means to support BI and environmental sensors. This classification has been done to optimize their required performance and processing cost of power and area. In the case of BI detection, while there have been several sensing methods, such as multi-frequency analysis, capacitive measurement, and current injection [12,13,14], this work selected the oscillator-based method to support other kinds of sensors. The oscillator-based path can provide direct digital conversion from resistive or capacitive sensor variations, called X-to-digital converters (XDCs), which substitutes conventional analog-to-digital converters (ADCs). Therefore, depending on each sensor’s operating path, the ADC or the XDC are selectively utilized for its digital conversion. For the ADC implementation, successive approximation register (SAR) types have been used widely in low-power applications due to their excellence in power efficiency compared with other ADC architectures. Conventional SAR ADCs consist of capacitive digital-to-analog converters (C-DACs), a dynamic comparator, and control logics, which do not require static power consumption. In order to improve noise immunity and signal-to-noise ratio (SNR), the SAR ADC is reformed to include a proposed scheme of alternate sampling (AS) and error correction.

The remainder of this paper is organized as follows. In Section 2, we present the proposed display-based multi-sensor interface architecture and describe the alternate-sampling error-correction scheme. Section 3 explains circuit implementation of the overall function following each sensor-signal path. Section 4 shows experiment results of its prototype design including the reconfigurable ROIC and AS-SAR ADC. Finally, the conclusion is given in Section 5.

## 2. Display-Based Multi-Sensor Interface

### 2.1. Heterogeneous Architecture

**Figure 1:**
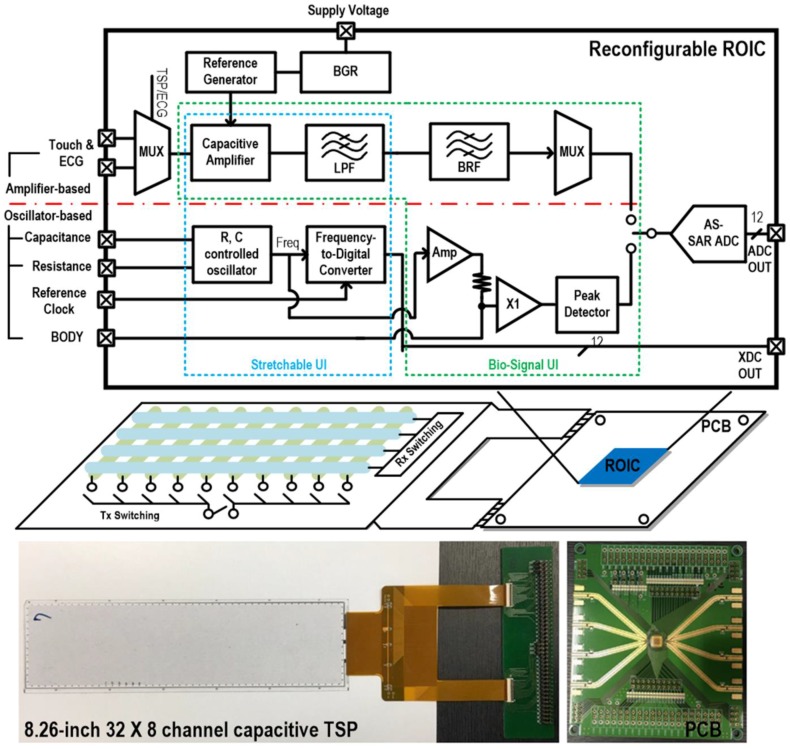
Display-based multi-sensor interface and reconfigurable readout integrated circuit.

Figure 1 presents a conceptual diagram of the proposed reconfigurable readout integrated circuit (ROIC) for heterogeneous sensors, also including a system prototype on a TSP that has 32 transmit (TX) lines and 8 receive (RX) lines. While the main role of the previous TSP interface was to detect various touch events, the proposed architecture utilizes the TSP itself as a kind of multi-purpose sensing channel, where different sensing functions are achieved depending on its readout circuit configuration. For effective implementation of the display-based multi sensor system, whole sensor readout topologies are consolidated into the following two basis readout circuits: amplifier-based and oscillator-based. That is, since most TSP readouts are implemented with capacitive amplifiers and weak ECG signals need to be amplified, they are merged together into a single amplifier-based readout circuit, utilizing the TSP cells as capacitive electrodes for the ECG operation. Remaining functions of BI and environmental sensors are unified into the oscillator-based readout circuit, considering that the one requires frequency generation and the other can be detected by using oscillators. 

Depending on their applications, these heterogeneous sensor functions are classified into two types: stretchable and bio-signal user interfaces (UIs). The stretchable UI can detect capacitive and resistive variations, and it can be utilized to support various user interfaces such as touch, stretching, or bending, especially in flexible display applications. Moreover, many environmental sensors have capacitive or resistive sensor characteristics [15]. Excluding the touch interface that uses the amplifier-based readout, these resistive and capacitive sensing interfaces are provided by making the oscillator-based readout frequency respond to variations of resistance or capacitance. Then, its digital conversion is done by utilizing a counter, and this operation is called the XDC, which does not require additional ADCs. The bio-signal UI is designed to provide physiological signals such as BI and ECG. The ECG interface is supported by the amplifier-based readout circuit, utilizing TSP cells as capacitive electrodes. For better capacitive signal coupling, the TSP cells are tied together to formulate bigger capacitance. The BI interface is provided by the oscillator-based readout circuit. Since the BI requires multiple frequency sweeps, the oscillator frequency is changed by programming internal control bits. For noise-immune design, the proposed ROIC includes an alternate-sampling successive approximation register (AS-SAR) ADC which has error-correction capability. Then, it is used for the digital conversion of the bio-signal UI and the touch interface, while the stretchable UI excluding the touch is supported by the XDC.

### 2.2. Alternate-Sampling Error-Correction Scheme

**Figure 2:**
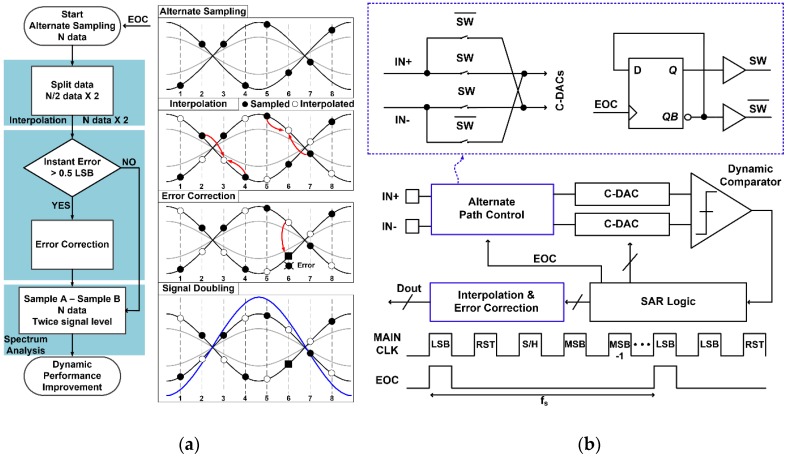
(**a**) Alternate-sampling error-correction scheme and (**b**) its circuit implementation.

Many studies have presented ways for achieving better resolution while maintaining the low-power characteristic of SAR ADC [16,17,18,19]. In order to compensate for mismatch and non-linearity in C-DACs, which mainly limit overall ADC resolution, various technologies including dithering [16] and digital-domain calibration [17] have been used. Hybrid architectures with integrating ADCs [18] or oversampling ADCs [19] have also been adopted to partially utilize high-resolution technologies. However, these previous works require complex additional circuits and supplementary conversion [20,21], and an alternate-sampling (AS) SAR ADC structure is proposed to relieve this difficulty. Figure 2a describes the proposed AS scheme in SAR ADCs and how to provide error-correction capability. The ADC circuit is designed to be fully differential, but its input path from various sensor outputs is single-ended. Therefore, the first role of the AS scheme is to convert the single-ended sensor input into its corresponding differential signal which can improve common-mode noise immunity considerably. The second role is that the converted differential data can be used for error correction by comparing it with an interpolated values from adjacent data. In this way, the proposed AS scheme can provide better common-mode noise immunity and also a new function of error correction.

The alternate sampling method is conceptually based on correlated double sampling (CDS) technology [22]. As shown in Figure 2b, it operates like conventional differential SAR ADCs, except that polarity of a differential input is alternately inverted by the end-of-conversion (EOC) signal. Two input paths are connected directly or crossly depending on their control signal switch (SW), and the input sampling polarity is inverted whenever the EOC signal is activated. That is, two differential inputs are then sampled onto C-DACs in phase or anti-phase, and then they are compared in a dynamic comparator whose output adjusts the next-cycle value of C-DACs. In this way, the successive approximation procedure of N cycles is performed to make the residue smaller than half of the least significant bit (LSB) value. After this N-cycle SAR conversion, N-bit ADC outputs are obtained and the EOC flag is activated for one cycle. The rising edge of the EOC inverts the polarity of the next differential input sampling, which is again reversed at the end of the next conversion period.

In this way, the proposed AS-SAR ADC performs periodic phase alternation in differential input sampling during conventional SAR conversion. Four detailed procedures are as follows. First, in-phase and anti-phase differential inputs are alternately sampled and converted to digital data, resulting in incomplete differential digital signals. Second, alternately vacant data in sampled differential waveforms are interpolated from adjacent sample points. Then, the interpolated values from in-phase sampled data can be converted into its corresponding anti-phase values which can be compared with anti-phase sampled data. Through these comparisons between the sample data and the interpolated values, instant errors in each sample can be detected as:

(1) $$D_{SampleA}\left(i\right)+D_{sampleB}\left(i\right)=\;D_{\max code}\left(i\right)+e_{instant},$$

where $D_{SampleA}\left(i\right)$ and $D_{sampleB}\left(i\right)$ are ith differentially-sampled data, and instant errors of $e_{instant}$ occur along to their peak-to-peak amplitude of $D_{\max code}\left(i\right)$. Without errors, sum of in-phase and anti-phase sampled data becomes equal to $D_{\max code}\left(i\right)$. The ADC has an average power of quantization noise V_LSB_^2^/12 and the magnitude of acceptable error is smaller than 0.5 LSB [23]. Then, if $e_{instant}$ is larger than 0.5 LSB, the erroneous sample can be corrected with its counterpart interpolated value that is estimated from its adjacent anti-phase samples. The proposed scheme can also correct consecutive errors thanks to the inherent splitting operation of the alternate sampling. If there are two consecutive errors, the proposed work can correct them by alternating two sampled values and comparing them with their corresponding interpolated values. As a final procedure, a signal doubling function is achieved by taking the difference of two anti-phased samples, which is inherently performed in differential SAR ADC conversion. While conventional differential signaling provides common-mode noise rejection and effective signal doubling (2×), the proposed scheme provides additional noise rejection and quadruple increments of effective signal strength (4×), resulting in meaningful SNR improvement. Considering the effects of offset cancellation and low-frequency rejection in the CDS technology, this proposed AS-SAR ADC is supposed to reduce input offset and its low-frequency noises.

## 3. ROIC Implementation

### 3.1. Design of Oscillator-Based Readout Circuit

**Figure 3:**
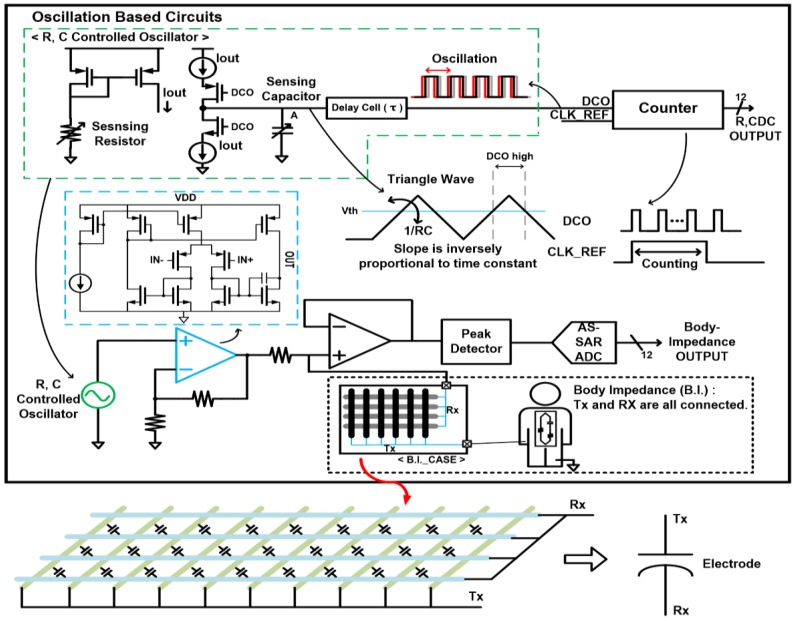
Oscillator-based multi-sensor interface configuration and readout circuit.

The proposed display-based multi-sensor readout implementations are consolidated into two base circuits: oscillator-based and amplifier-based. First, the oscillation-based readout interface that is shown Figure 3 is selectively configured to utilize a resistance- or capacitance-controlled oscillator as the XDC for resistive/capacitive sensors or the signal source for the BI measurement. The oscillator consists of a charge pump and a delay cell, and its charging/discharging speed can be controlled by load capacitance or by its current-mirror reference current. After some logic delay (τ), feedback connection of digitally controlled oscillator (DCO) constitutes a king of ring oscillator [24], and it converts the charging/discharging speed movement into the frequency displacement which can be converted to digital values through the counting process against a fixed-frequency external clock (CLK_REF). In this way, the proposed oscillator-based interface can work as the XDC and also readout capacitive or resistive sensor variations, without requiring additional ADCs.

On the other hand, the BI measurement is performed by measuring body impedance at multiple frequencies that are available from the same oscillator as in the XDC. Like multi-frequency bioelectrical impedance analysis [12], low frequency ranges of 1 k to 500 kHz are used for the BI measurement. The human body consists of resistance and capacitance from the cell membranes, and the body model is composed of their parallel combination. The lower part of Figure 3 presents the BI measurement configuration that utilizes TSP cells as capacitive electrodes. Since each TSP cell has small capacitance and contact area, whole TSP cell’s TX/RX electrodes are merged together to maximize the mutual capacitance and possible contact area. The electrical characteristic that passes through the human body is measured by a peak detector and then converted to digital value by the AS-SAR ADC.

### 3.2. Design of Amplifier-Based Readout Circuit

**Figure 4:**
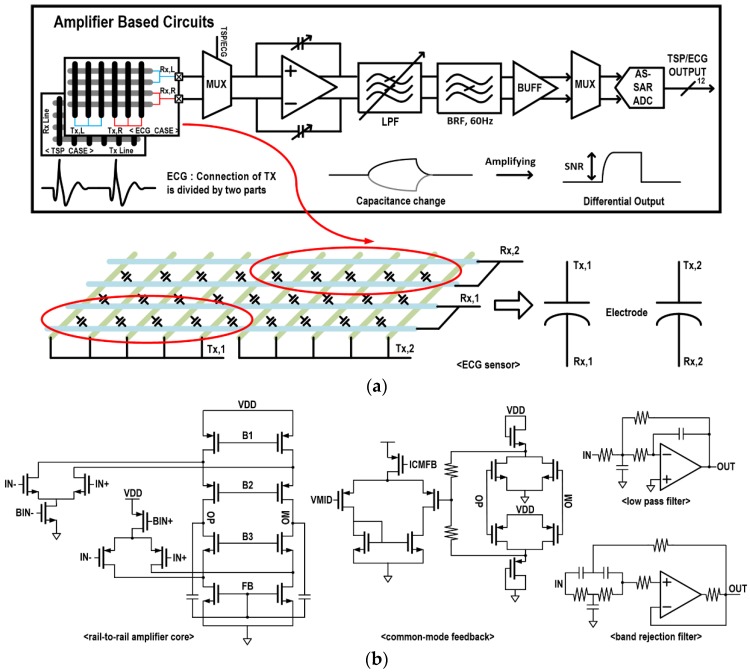
(**a**) Amplifier-based multi-sensor interface configuration; and (**b**) readout circuits.

Figure 4 presents the amplifier-based multi-sensor configuration and its reconfigurable readout circuit for touch and ECG. For the touch operation, touch events are converted to capacitance variations through the TSP, and these capacitive changes are sensed by adjusting the capacitive amplifier’s gain on mutually coupled signals from the TX signal. For compact design of the ROIC, every TSP cell is multiplexed and supported by a single capacitive amplifier. For high-precision touch detection, it is followed by a low-pass filter (LPF) to remove various environmental noises. A band-reject filter (BRF) is selectively used to remove power-supply noise of 50 or 60 Hz [25]. 

For the ECG detection, the TSP is configured to provide two capacitive electrodes by dividing the panel by half and merging the two resulting half-panel’s TX and RX lines, as shown in the lower part of Figure 4. Considering 300 Hz bandwidth of the ECG signal [26], the LPF bandwidth is adjusted through programming switches. After the BRF and additional amplification, its digital conversion is done by the 12-bit AS-SAR ADC. In this way, a two sensor interface for the touch and the ECG are effectively supported by utilizing a single amplifier-based readout circuit, thus, minimizing their area and power consumption. The capacitive amplifier is designed to support rail-to-rail operation and to be fully differential for noise immunity. Its detailed schematics with common-mode feedback is shown in Figure 4b, where schematics of the LPF and the BRF are also included.

### 3.3. Design of 12-Bit Alternate-Sampling SAR ADC

**Figure 5:**
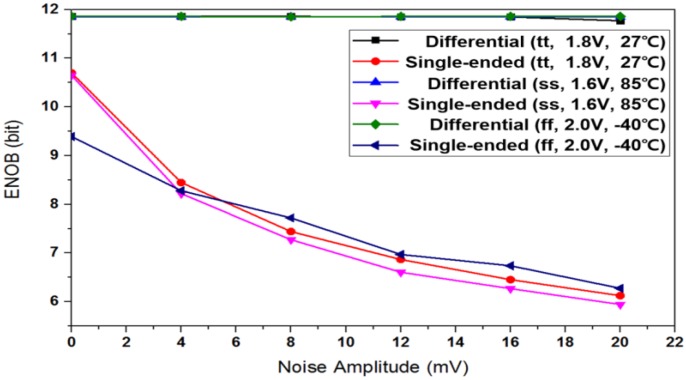
Spectrum analysis of successive approximation register (SAR) analog-to-digital converters (ADC) architectures with random noise.

Most sensor interfaces are single-ended so that they are weak to various noises and fluctuations, even though the following ADC circuit is fully differential. Figure 5 shows circuit-level process-corner simulation results about how much the effective number of bits (ENOB) degrades depending on the input-stage structure of either single-ended or differential input when common-mode noises at SAR ADC inputs become stronger. While conventional single-ended input structure is easily corrupted, differential input structure is very immune to the common-mode noises. This is one of the motivations to propose the AS technique which generates differential signals at single-ended sensor interfaces. Another benefit of the AS is the capability of error correction as shown in Figure 2. Therefore, a 12-bit AS-SAR ADC is used in high-resolution sensor interfaces of touch, ECG, and BI, while it is replaced with the XDC in cases of the other sensor interfaces. 

**Figure 6:**
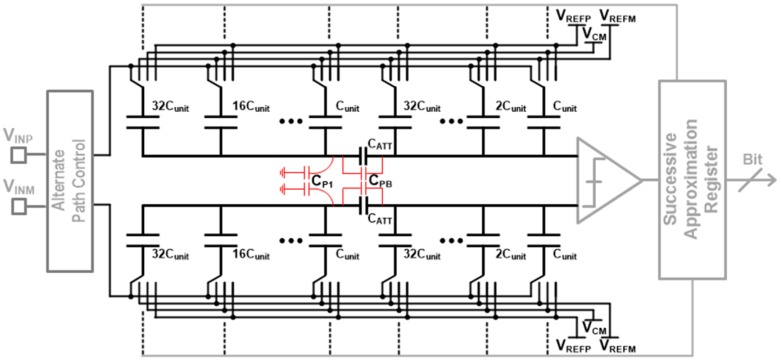
Binary weighted split capacitive digital-to-analog converters (C-DACs).

Figure 6 shows a 12-bit fully differential AS-SAR ADC, where binary-weighted C-DACs adopt the split capacitor structure for smaller areas and better matching. The C-DAC array is split into two identical sub-DACs by the attenuation capacitor ($C_{ATT}$) which is given by

(2) $$C_{ATT}=\frac{Total\;capacitance\;of\;MSB\;parts}{Total\;capacitance\;of\;LSB\;parts}\;$$

In case of 12-bit implementation, $C_{ATT}$ becomes (64/63)* $C_{unit}$. In Figure 6, the right side of $C_{ATT}$ is of MSB (most significant bit) part and the left side is of LSB (least significant bit) part. If the $C_{ATT}$ value is exactly the same as in (2), series combination of the LSB part and $C_{ATT}$ is equivalent to unit capacitance ($C_{unit})$. However, its real implementation is hard to match exactly because of parasitic effects. So, its revised equivalent capacitance with parasitic effects is given by

(3) $$C_{Eq}=\frac{\left(C_{ATT}+C_{P1}\right)\times 2^{k}\cdot C_{unit}}{2^{k}\cdot C_{unit}+C_{P1}+C_{ATT}+C_{PB}}$$

where $C_{PB}$ is parasitic capacitance between the MSB part and the LSB part and $C_{P1}$ is from the switched-capacitor array and its layout routings in the LSB part. Since $C_{P1}$ critically degrades the ADC accuracy, its effect is minimized by optimizing the $C_{ATT}$ value through post-layout simulations. 

## 4. Measurement Results & Discussions

**Figure 7:**
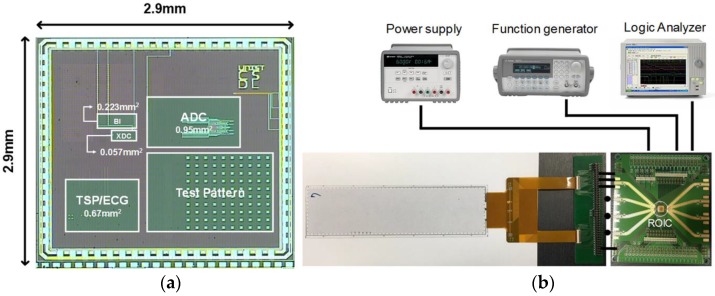
Proposed reconfigurable readout integrated circuit (ROIC) prototype: (**a**) microphotograph; (**b**) measurement setup.

**Figure 8:**
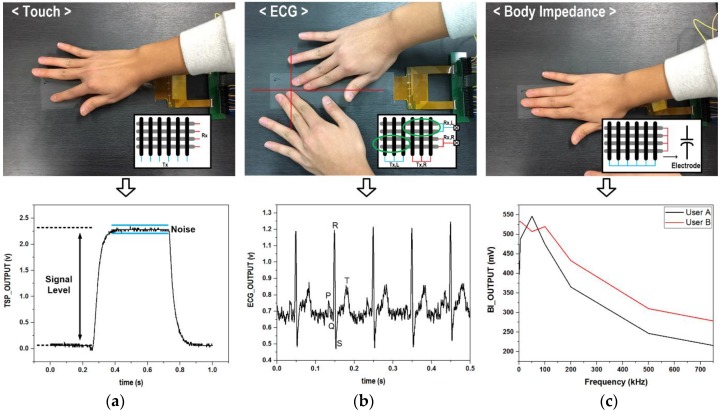
Measurement results of touch screen panel (TSP)-based sensing functions: (**a**) touch; (**b**) electrocardiogram (ECG); and (**c**) body impedance.

The proposed multi-sensor ROIC prototype was fabricated in a 0.18 μm complementary metal oxide semiconductor (CMOS), and Figure 7a shows its microphotograph whose chip size is 2.9 mm × 2.9 mm. The core area is 1.9 mm^2^, where the oscillator-based ROIC, the amplifier-based ROIC, and the AS-SAR ADC occupy 0.28 mm^2^, 0.67 mm^2^, 0.95 mm^2^, respectively. Figure 7b shows a display-based multi-sensor prototype and its measurement setup, where an 8.26-inch 32 × 8 channel capacitive TSP was utilized. For implementation of five heterogeneous sensing functions, the reconfigurable multi-sensor interface was configured as the TSP-based detection mode and the environmental detection mode. Figure 8 shows measurement results of the TSP-based detection mode for the touch, the ECG, and the BI. The touch function was verified to achieve a signal-to-noise ratio (SNR) of 44.36 dB through finger-touch operation as shown in Figure 8a. The ECG was measured by placing two hands onto two different half-planes of the TSPs, and its measured ECG waveform is shown in Figure 8b. For the BI measurement, all TSP pixels were utilized to maximize the capacitive amplifier gain, and Figure 8c shows measured frequency characteristics. 

**Figure 9:**
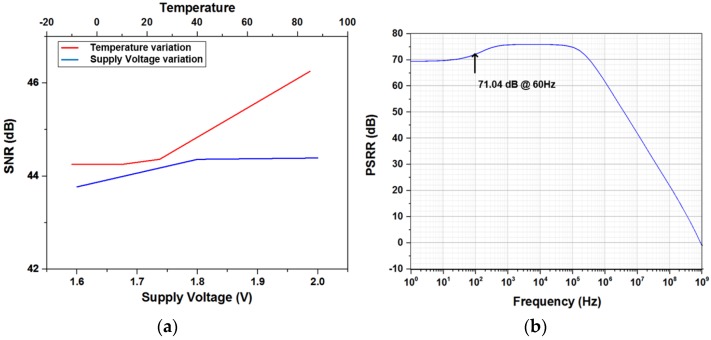
(**a**) Measured ROIC signal-to-noise ratios (SNRs) over temperature and supply-voltage variations; (**b**) ROIC power-supply rejection ratio **(**PSRR) (simulated).

**Figure 10:**
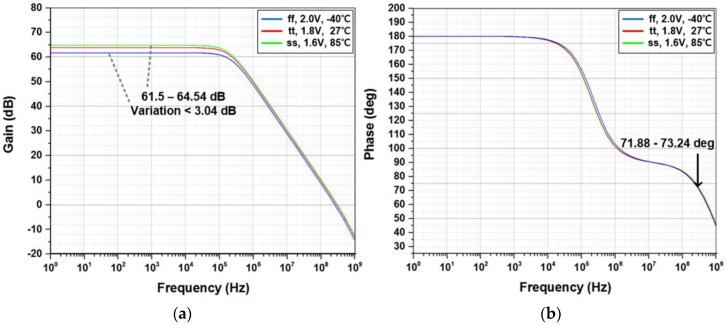
Fully-differential amplifier characteristic over process-voltage-temperature (PVT) variations (simulated): (**a**) gain (**b**) phase margin.

**Figure 11:**
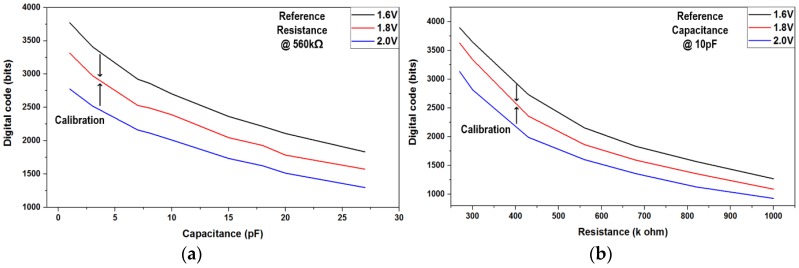
Measured characteristics of X-to-digital converters (XDC)-based readout circuit: (**a**) capacitive and (**b**) resistive.

Figure 9a gives the ROIC characteristic responses in the amplifier-based configuration when supply voltage and temperature are swept from 1.6 V to 2.0 V and from −12 ℃ to 85 ℃, respectively. Its measured SNR, especially in the touch sensing mode, improved slightly with respect to increments of supply and temperature. This improvement is supposed to be caused by gain increments of the differential amplifier whose process-corner simulation results are given in Figure 10. Figure 9b shows the power-supply rejection ratio (PSRR) of the amplifier-based readout circuit. Due to its fully-differential implementation, it achieved the PSRR of 71 dB at 60 Hz where a lot of power-supply noise appears. The XDC detection mode for capacitive and resistive sensors was functionally verified by utilizing external resistors and capacitors. Figure 11 shows two measured sensing characteristics by utilizing the single oscillator-based XDC circuit, where it supports the capacitive sensing range from 0 pF to 27 pF and the resistive range of from 270 kΩ to 1 MΩ. The effect of supply-voltage variation on the capacitive and resistive sensor characteristics are also included together, which means that an additional supply-voltage detection circuit might calibrate the corresponding sensor characteristic variation.

**Figure 12:**
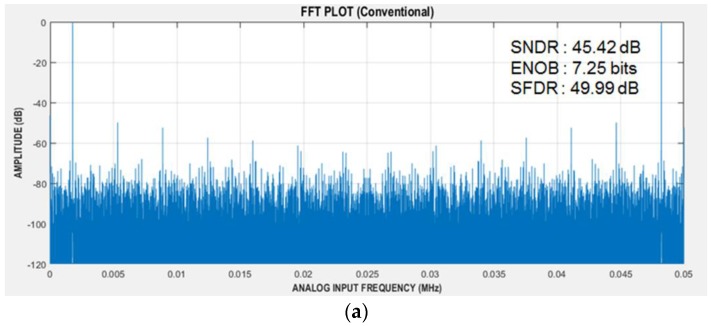

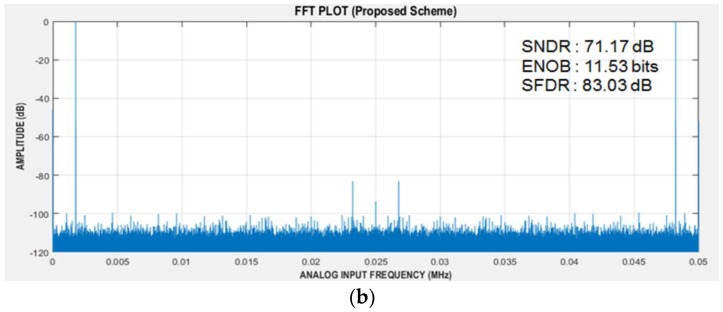
Measured 262144-point fast Fourier transform (FFT) spectrums at 1.777 kHz input (**a**) with (**b**) without alternate sampling and error correction scheme.

The AS-SAR ADC, which is commonly used for the TSP-based sensors, was integrated together in the ROIC, but post-processing algorithms of the interpolation and error correction were implemented in the MATLAB. Figure 12 compares the measured fast Fourier transform (FFT) spectrums before and after applying the proposed alternate-sampling and error-correction scheme. The measured signal-to-noise and distortion ratio (SNDR) was improved from 45.2 dB to 71.17 dB, and the measured effective number of bits (ENOB) was from 7.25 bits to 11.53 bits. The measured spurious-free dynamic range (SFDR) was also improved from 49.99 dB to 83.03 dB. In this AS-SAR ADC, the C-DACs utilized 70 fF metal-insulator-metal (MIM) devices as unit capacitors to meet the kT/C noise requirement.

**Figure 13:**
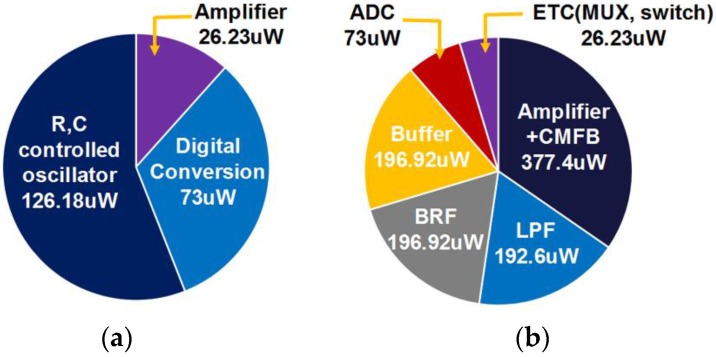
Power breakdown of the sub-blocks: (**a**) oscillator-based circuits and (**b**) amplifier-based circuits.

Figure 13 shows the power breakdown of sub-blocks in the proposed reconfigurable ROIC, where their average power consumption is given depending on their operational configuration. In the case of the oscillator-based operations, the oscillator and the single-ended amplifier consume 126.18 μW and 26.23 μW, respectively. The digital conversion requires about 73 μW. In the amplifier-based operation mode, differential amplifiers and filters for better noise immunity increase the ROIC power consumption. Total power consumption is 0.23 mW for the oscillator-based mode and 1.09 mW for the amplifier-based mode, resulting in optimization of the power consumption for its operational modes.

This work was compared with recent works, which are summarized in Table 1. The proposed multi-sensor ROIC was experimentally verified to support five heterogeneous sensing functions while most previous works provide only one or two sensing functions. Thanks to the proposed reconfigurable ROIC structure, wide ranges of sensor capacitance and resistance are simultaneously supported, and the TSP interface itself provides competitive performance of the SNR with smaller area and power consumption. In the proposed reconfigurable ROIC structure, three TSP-based sensing circuits were implemented to reduce the area and the power consumption by selectively reusing the single readout circuit for all 32 × 8 TSP pixels and also by utilizing the TSPs as capacitive electrodes. The ADC was designed to have 12-bit resolution which is sufficient for most sensing functions, including the ECG and the TSPs. The environmental sensing functions were implemented by utilizing the same readout circuit without additional area, and its low-power characteristic was achieved through the XDC operation which does not need to activate the ADC.

## 5. Conclusions

This paper presented a reconfigurable readout integrated circuit with the following two operation modes: amplifier-based and the oscillator-based. Its system prototype of the display-based multi-sensor interface was implemented and experimentally verified to support five sensor types: touch, ECG, BI, resistive, and capacitive environmental sensors. By making sophisticated changes to the TSP interface configuration, the three sensory interfaces of touch, ECG, and BI were supported. The oscillator-based operation mode was utilized to support resistive and capacitive environmental sensors and stretchable interfaces. The AS scheme, embedding differential conversion, signal doubling, and error correction, was proposed to achieve better effective resolution and noise immunity against inherent human-touch operations. A 12-bit AS-SAR ADC was integrated in the ROIC and the proposed AS scheme was experimentally verified to provide about 4-bit improvement of the ENOB. Other characteristics of SNDR and SFDR were improved from 45.42 dB to 71.17 dB and from 49.99 dB to 83.03 dB, respectively. This reconfigurable work was designed to provide heterogeneous multi-sensor interfaces in multiple applications of display systems, flexible electronics, and wearable devices. Potentially, the bio-signal interface can be utilized in healthcare devices, and also the XDC interface can be applied to various environmental monitoring systems. The AS scheme can correct two consecutive errors, and further correction capability will be provided through upcoming work.

## Figures and Tables

**Table 1:** Performance summary and comparison with recent works.

Parameter	This Work	[1]	[9]	[10]	[11]
Process	0.18 µm	0.18 µm	0.18 µm	0.16 µm	0.18 µm
Supported sensor types	TSP, R/C type, body impedance (BI), ECG	TSP	BI, ECG	C type	R type
Power consumption (mW)	1.163 (TSP/ECG + ADC) 0.202 (XDC) 0.271 (BI + ADC)	6.26 (TSP)	0.233 (3 channel BI + ECG )	0.014 (capacitance-to-digital converter (CDC))	0.0017 (resistance-to-digital converter (RDC))
Area (mm^2^)	0.67 (TSP/ECG) 0.057 (XDC) 0.223 (BI) 0.95 (ADC)	2.2	8.17 (estimated per channel) 49 (ROIC + micro controller unit (MCU))	0.05	1.23
Application	Bio-signal acquisition, TSP, R & C type sensor	TSP	Bio-signal acquisition	C type sensor	R type sensor
Supply (V)	1.8	2.1–3.3	1.2	1	1.2/0.6
Resolution (bits)	12	N.A.	13.5	13.1 (CDC)	10
R, C sensing Range (Ω), (F)	R: 270 k–1 M C: 0 p–27 p	N.A.	N.A.	C: 0–8 p	R: 10 k–10 M
TSP channel	TX: 32 RX: 8	TX: 12 RX: 8	N.A.	N.A.	N.A.
Frame rate (Hz)	240	50–6400	N.A.	N.A.	N.A.
TSP SNR (dB)	44.36	40 @ oversampling ratio (OSR) 32	N.A.	N.A.	N.A.
Body impedance frequency range (Hz)	50 k–750 k	N.A.	1–20 M	N.A.	N.A.
